# Supplementary material for: Pyruvate Dehydrogenase Kinase 1 inhibition mediated oxidative phosphorylation enhancement in cartilage promotes osteoarthritis progression
Source: BMC Musculoskelet Disord. 2023 Jul 20;24:597. doi: 10.1186/s12891-023-06585-6 (PMC10357736; doi:10.1186/s12891-023-06585-6)
Supplement: Supplementary file 1 — Additional file 1. [file 12891_2023_6585_MOESM1_ESM.zip › BP.Gsea.1653623667859/GOBP_RENAL_SYSTEM_VASCULATURE_DEVELOPMENT.html]

Details for gene set GOBP\_RENAL\_SYSTEM\_VASCULATURE\_DEVELOPMENT[GSEA]

|  || Dataset | 55457\_collapsed\_to\_symbols.cls.cls #OA\_versus\_normal.cls.cls #OA\_versus\_normal\_repos |
| Phenotype | cls.cls#OA\_versus\_normal\_repos |
| Upregulated in class | normal |
| GeneSet | GOBP\_RENAL\_SYSTEM\_VASCULATURE\_DEVELOPMENT |
| Enrichment Score (ES) | -0.53858256 |
| Normalized Enrichment Score (NES) | -1.8290175 |
| Nominal p-value | 0.0035149385 |
| FDR q-value | 0.08096122 |
| FWER p-Value | 0.945 |
Table: GSEA Results Summary

  

Fig 1: Enrichment plot: GOBP\_RENAL\_SYSTEM\_VASCULATURE\_DEVELOPMENT      
 Profile of the Running ES Score & Positions of GeneSet Members on the Rank Ordered List

  

| SYMBOL | TITLE | RANK IN GENE LIST | RANK METRIC SCORE | RUNNING ES | CORE ENRICHMENT || 1 | ANGPT1 | angiopoietin 1 [Source:HGNC Symbol;Acc:HGNC:484] | 1467 | 0.433 | -0.0696 | No |
| 2 | PDGFRA | platelet derived growth factor receptor alpha [Source:HGNC Symbol;Acc:HGNC:8803] | 2943 | 0.256 | -0.1580 | No |
| 3 | NRP1 | neuropilin 1 [Source:HGNC Symbol;Acc:HGNC:8004] | 3704 | 0.185 | -0.1981 | No |
| 4 | PDGFD | platelet derived growth factor D [Source:HGNC Symbol;Acc:HGNC:30620] | 3850 | 0.171 | -0.1919 | No |
| 5 | TCF21 | transcription factor 21 [Source:HGNC Symbol;Acc:HGNC:11632] | 4321 | 0.127 | -0.2154 | No |
| 6 | CD34 | CD34 molecule [Source:HGNC Symbol;Acc:HGNC:1662] | 4341 | 0.125 | -0.2042 | No |
| 7 | ACTA2 | "actin alpha 2, smooth muscle [Source:HGNC Symbol;Acc:HGNC:130]" | 4490 | 0.114 | -0.2040 | No |
| 8 | EDNRA | endothelin receptor type A [Source:HGNC Symbol;Acc:HGNC:3179] | 4663 | 0.099 | -0.2072 | No |
| 9 | WT1 | WT1 transcription factor [Source:HGNC Symbol;Acc:HGNC:12796] | 5452 | 0.037 | -0.2647 | No |
| 10 | PKD2 | "polycystin 2, transient receptor potential cation channel [Source:HGNC Symbol;Acc:HGNC:9009]" | 6254 | -0.024 | -0.3244 | No |
| 11 | PDGFRB | platelet derived growth factor receptor beta [Source:HGNC Symbol;Acc:HGNC:8804] | 6914 | -0.073 | -0.3681 | No |
| 12 | SERPINB7 | serpin family B member 7 [Source:HGNC Symbol;Acc:HGNC:13902] | 7679 | -0.133 | -0.4138 | No |
| 13 | BMP4 | bone morphogenetic protein 4 [Source:HGNC Symbol;Acc:HGNC:1071] | 7816 | -0.142 | -0.4098 | No |
| 14 | NOTCH3 | notch receptor 3 [Source:HGNC Symbol;Acc:HGNC:7883] | 7894 | -0.147 | -0.4008 | No |
| 15 | TEK | TEK receptor tyrosine kinase [Source:HGNC Symbol;Acc:HGNC:11724] | 8623 | -0.203 | -0.4366 | No |
| 16 | BMP7 | bone morphogenetic protein 7 [Source:HGNC Symbol;Acc:HGNC:1074] | 9544 | -0.278 | -0.4796 | No |
| 17 | PECAM1 | platelet and endothelial cell adhesion molecule 1 [Source:HGNC Symbol;Acc:HGNC:8823] | 10304 | -0.351 | -0.5027 | Yes |
| 18 | ANGPT2 | angiopoietin 2 [Source:HGNC Symbol;Acc:HGNC:485] | 10401 | -0.361 | -0.4732 | Yes |
| 19 | NOTCH2 | notch receptor 2 [Source:HGNC Symbol;Acc:HGNC:7882] | 10720 | -0.395 | -0.4574 | Yes |
| 20 | IL6R | interleukin 6 receptor [Source:HGNC Symbol;Acc:HGNC:6019] | 11615 | -0.519 | -0.4738 | Yes |
| 21 | EGR1 | early growth response 1 [Source:HGNC Symbol;Acc:HGNC:3238] | 12052 | -0.607 | -0.4455 | Yes |
| 22 | CFLAR | CASP8 and FADD like apoptosis regulator [Source:HGNC Symbol;Acc:HGNC:1876] | 12079 | -0.614 | -0.3847 | Yes |
| 23 | PDGFA | platelet derived growth factor subunit A [Source:HGNC Symbol;Acc:HGNC:8799] | 12104 | -0.621 | -0.3230 | Yes |
| 24 | FOXC2 | forkhead box C2 [Source:HGNC Symbol;Acc:HGNC:3801] | 12218 | -0.650 | -0.2652 | Yes |
| 25 | PDGFB | platelet derived growth factor subunit B [Source:HGNC Symbol;Acc:HGNC:8800] | 12389 | -0.704 | -0.2064 | Yes |
| 26 | NOTCH1 | notch receptor 1 [Source:HGNC Symbol;Acc:HGNC:7881] | 12555 | -0.776 | -0.1398 | Yes |
| 27 | GPR4 | G protein-coupled receptor 4 [Source:HGNC Symbol;Acc:HGNC:4497] | 12561 | -0.778 | -0.0606 | Yes |
| 28 | HES1 | hes family bHLH transcription factor 1 [Source:HGNC Symbol;Acc:HGNC:5192] | 12676 | -0.849 | 0.0174 | Yes |
Table: GSEA details [plain text format]

  

Fig 2: GOBP\_RENAL\_SYSTEM\_VASCULATURE\_DEVELOPMENT      
 Blue-Pink O' Gram in the Space of the Analyzed GeneSet

  

Fig 3: GOBP\_RENAL\_SYSTEM\_VASCULATURE\_DEVELOPMENT: Random ES distribution      
 Gene set null distribution of ES for **GOBP\_RENAL\_SYSTEM\_VASCULATURE\_DEVELOPMENT**
